# Comparison of nonparametric methods for static visual field interpolation

**DOI:** 10.1007/s11517-016-1485-x

**Published:** 2016-04-22

**Authors:** Travis B. Smith, Ning Smith, Richard G. Weleber

**Affiliations:** 1 Casey Eye Institute, Oregon Health & Science University, 3375 SW Terwilliger Blvd., Portland, OR 97239-4197 USA; 2 Center for Health Research, Kaiser Permanente, Portland, OR USA

**Keywords:** Interpolation, Visual fields, Perimetry, Retinitis pigmentosa

## Abstract

Visual field testing with standard automated perimetry produces a sparse representation of a sensitivity map, sometimes called the hill of vision (HOV), for the retina. Interpolation or resampling of these data is important for visual display, clinical interpretation, and quantitative analysis. Our objective was to compare several popular interpolation methods in terms of their utility to visual field testing. We evaluated nine nonparametric scattered data interpolation algorithms and compared their performances in normal subjects and patients with retinal degeneration. Interpolator performance was assessed by leave-one-out cross-validation accuracy and high-density interpolated HOV surface smoothness. Radial basis function (RBF) interpolation with a linear kernel yielded the best accuracy, with an overall mean absolute error (MAE) of 2.01 dB and root-mean-square error (RMSE) of 3.20 dB that were significantly better than all other methods (*p* ≤ 0.003). Thin-plate spline RBF interpolation yielded the best smoothness results (*p* < 0.001) and scored well for accuracy with overall MAE and RMSE values of 2.08 and 3.28 dB, respectively. Natural neighbor interpolation, which may be a more readily accessible method to some practitioners, also performed well. While no interpolator will be universally optimal, these interpolators are good choices among nonparametric methods.

## Introduction

Interpolation of static visual field sensitivity data offers several benefits. By resampling examination data onto a single common grid, it can unify disparate perimetry protocols. This facilitates the joint analysis of examinations conducted under different studies or with different test grid patterns, which otherwise would be difficult to compare. Interpolation is also useful for transforming data from irregular or sparse grids into a uniformly sampled format. Resampling onto a high-density regular grid produces a surface representation of the hill of vision (HOV) which offers more flexibility in visualizing examination results than conventional display methods that show only discrete numerical or coarsely quantized information [15, 26]. Surface renderings of the HOV are helpful to clinicians not only in disease monitoring and treatment, but also in providing a visual model to aid in discussions of examination results with patients [13]. Furthermore, densely sampled HOV surfaces enable new quantitative analysis techniques and new clinical trial end points. For example, topographic interpretation of interpolated HOV surfaces supports contour-based and volumetric approaches to visual field analysis [27].

Here, we compare various interpolators to help identify the best methods for static visual field data. Similar interpolator comparisons have been made in other areas of study (for example, in mathematics [10], geology [4], and climatology [23]). So far, however, only preliminary work has been done regarding visual fields [22]. In this study, we extend that work to include performance assessments of smoothness in addition to accuracy. Furthermore, the interpolators in this study were chosen by their suitability for clinical research and development. We limited our investigation to scattered data techniques, which can accommodate any test grid pattern. Also, each method was either strictly nonparametric or, if not, implemented with a fixed, standardized parameter value to eliminate complications associated with parameter estimation. We compared their performances with visual field data from a group of healthy, normal volunteers and patients with retinitis pigmentosa (RP), a family of inherited retinal degenerations that is characterized by progressive visual field loss [14]. The best-performing interpolators are identified, and their relative merits are discussed in relation to the performance metrics. As one of the first quantitative interpolation comparisons for visual fields, the results from this study will be useful to clinicians and researchers looking for validated interpolation strategies for visual field data.

## Methods

### Static perimetry data

**Fig. 1:**
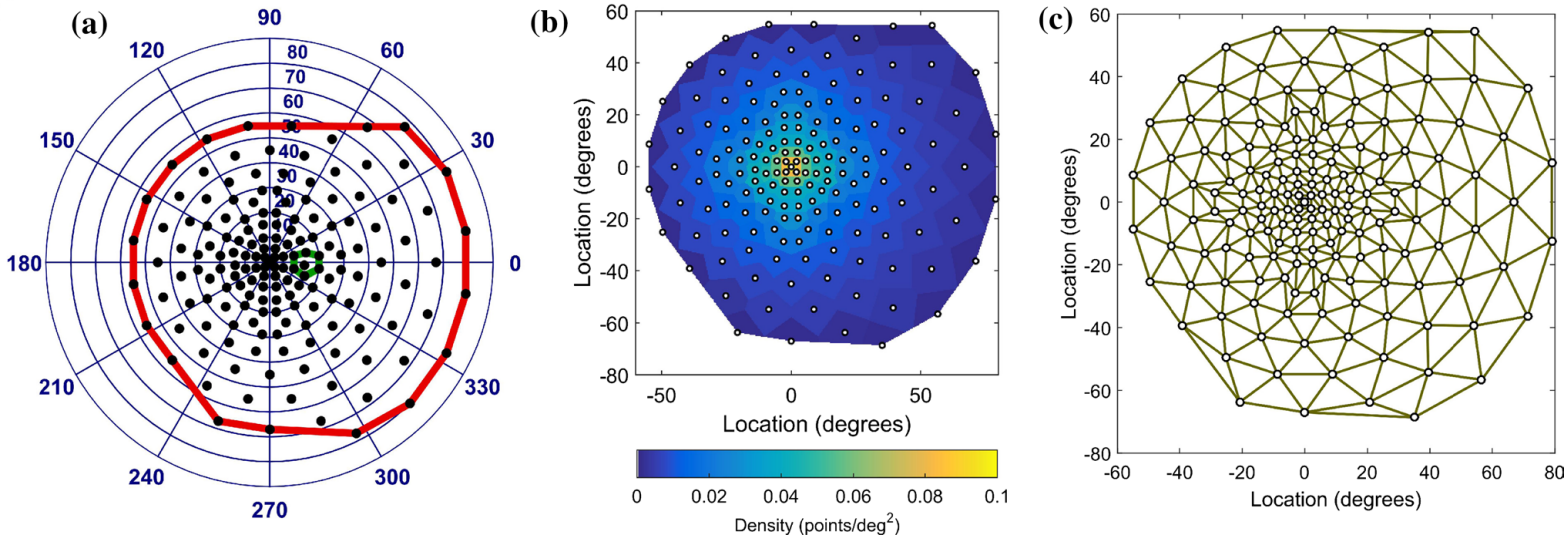
Details of the perimetry test grid pattern. **a** Sample points (*black circles*), convex hull (*red line*), and expected location of the optic disk and natural blind spot (*green line*) for the right eye. The grid spans 135° horizontally and 125° vertically. **b** Voronoi diagram of the sampling density showing the central condensation of the pattern. **c** Delaunay triangulation of the grid pattern

Full-field standard automated perimetry was performed on 10 normal subjects and 10-RP patients with an Octopus 101 perimeter (Haag-Streit AG, Köniz, Switzerland). The testing protocol used a 10 cd/m^2^ background luminance, the GATE-i fast thresholding strategy [20], and a size V (64 mm^2^) stimulus with 200-ms duration. Visual fields were tested with a binocularly symmetric, radially oriented, and centrally condensed grid pattern consisting of 164 test points (Fig. 1). The maximum allowable reliability factor (the percentage of catch trials that generated either a false-positive or a false-negative response) was 15 % for each normal examination and 25 % for each patient examination [27]. Replicate testing was performed within 90 days of the first test to obtain repeated measurements for each eye. Table 1 presents the mean age in each subject group and the distributions of repeated examinations.

**Table 1:** Summary of visual field data

ID	Gender	Age (years)^a^	Number of examinations (OD, OS)
N1	F	31.5	4 (2, 2)
N2	F	22.0	6 (3, 3)
N3	F	18.5	4 (2, 2)
N4	F	34.3	6 (3, 3)
N5	F	51.1	4 (2, 2)
N6	M	34.5	4 (2, 2)
N7	F	30.5	6 (3, 3)
N8	F	34.7	4 (2, 2)
N9	F	57.1	4 (2, 2)
N10	F	56.1	4 (2, 2)
P1	M	37.9	10 (5, 5)
P2	M	33.7	10 (5, 5)
P3	F	66.9	8 (4, 4)
P4	M	33.2	8 (4, 4)
P5	F	39.7	4 (2, 2)
P6	M	46.4	10 (5, 5)
P7	F	43.6	8 (4, 4)
P8	F	28.3	10 (5, 5)
P9	F	37.4	7 (4, 3)
P10	F	62.3	8 (4, 4)
All 10 normals	M: 1, F: 9	37.0 ± 13.5	46 (23, 23)
All 10 patients	M: 4, F: 6	42.9 ± 12.6	83 (42, 41)

*N* normal, *P* patient, *M* male, *F* female, *OD* right eye, *OS* left eye

^a^Based on the time of the first examination

Approval adhering to the tenets of the Declaration of Helsinki was obtained from the Oregon Health and Science University Institutional Review Board, and written informed consent was provided by all subjects.

### Interpolation methods

Each perimetry examination produced a 3-D point cloud of triplets $\left\{ {\left( {x_{i} ,y_{i} ,z_{i} } \right)} \right\}_{i = 1}^{N}$ with $N = 164$ test points where each point was represented by a retinal location $\left( {x_{i} ,y_{i} } \right)$ in angular coordinates and differential luminance sensitivity $\left( {z_{i} } \right)$, which was the light sensitivity in dB measured at that location. As seen in Fig. 1, the test points were irregularly spaced, necessitating scattered data interpolation methods.

We evaluated the 9 interpolation algorithms listed in Table 2. Each algorithm is a pure interpolator in that it reproduced the original data exactly, with no modeling, fitting, or approximation. Four of the interpolators were implemented with the built-in grid data function in MATLAB 8.5 (MathWorks, Natick, MA, USA). The other five interpolators, including both radial basis function methods, were implemented with custom software in MATLAB.

**Table 2:** Interpolation algorithms

Interpolator	Abbreviation	References
Nearest neighbor^a^	NN	[1]
Linear^a^	Lin	[1]
Cubic^a^	Cub	[1]
Natural neighbor^a^	NatN	[1]
Inverse distance weighting	IDW	[17]
Improved inverse distance weighting	IDW2	[9]
Linear radial basis function	RBFlin	[6]
Thin-plate spline radial basis function	RBFtps	[20]
Ordinary Kriging	Krig	[13]

^a^Based on Delaunay triangulation

The grid data-based methods are each related to the Delaunay triangulation [9] of the grid pattern (Fig. 1c), which produces a piecewise triangular network with nodes set to the original data point cloud. From this network, the interpolated value can be computed from either: the nearest node in the network for nearest neighbor (NN) interpolation, the linear plane containing the three nearest nodes for linear (Lin) interpolation, or the bivariate cubic polynomial passing through those three nodes for cubic (Cub) interpolation [1]. The fourth method was natural neighbor (NatN) interpolation, which is based on the Voronoi tessellation of the test points—the geometric dual to the Delaunay triangular network. Of these four methods, only cubic and natural neighbor interpolations are guaranteed to be continuous across the boundaries of the triangular network [1]; consequently, the linear and nearest neighbor interpolators will tend to have lower smoothness characteristics.

Two of the methods we analyzed were based on inverse distance weighting, also known as Shepard’s method. Here, each interpolated value is a weighted combination of every examination data point. In the classical formulation (IDW), the weights are inversely proportional to power of the distances to the data points [21]. In this study, we used the traditional square distance (power = 2) to balance the influence of points far away (emphasized with a small power) and nearby (emphasized with a large power). We also included the modified formulation of this interpolator (IDW2), which improves the localization by only including data points if they are within a specified distance from the interpolation location [11]. For this distance, we used the mean separation between all locations in the test grid, which was 48.3°.

Two of the methods are radial basis function (RBF) interpolators, which form each interpolated value through a linear combination of kernel functions. The kernels have the form $\phi \left( r \right)$ and are dependent only on the (radial) distance between the output location and all examination data locations. The linear weights are determined by solving a data-dependent system of equations. We considered two types of nonparametric kernels: linear, $\phi \left( r \right) = r$ [7], and thin-plate spline, $\phi \left( r \right) = r^{2} \log r$ [24].

In ordinary Kriging (Krig) or Gaussian process regression, each interpolated value is a weighted combination where the weights are derived from spatial covariances [17]. This stochastic approach treats the data as a random field consisting of mean and residual values at each location. This method is natively parametric, but the parameters can be estimated automatically through optimization to fit a covariance function that models the residuals in each examination. Here, we considered an exponential model, which is appropriate for data with an exponentially decaying spatial autocorrelation [25].

There are many other scattered data interpolation methods available. These 9 methods were chosen for this analysis because they are commonly used and frequently appear in other comparison studies [4, 8, 10, 23]. They are likely to be more easily available to researchers in the field than other esoteric methods, and they run relatively quickly on modern computers.

### Performance evaluation

We evaluated the interpolators in terms of accuracy and the smoothness of the high-density interpolated HOV surfaces. Greater smoothness indicates a lower prevalence of undesired behaviors such as surface discontinuities and peakiness. To assess accuracy, we generated a “truth” data set $\left\{ {\left( {x_{i} ,y_{i} ,\tilde{z}_{i} } \right)} \right\}_{i = 1}^{N}$ for each eye by averaging the sensitivity values across all examinations for that eye. Here,

1 $$\tilde{z}_{i} = \frac{1}{{N_{e} }}\mathop \sum \limits_{j = 0}^{{N_{e} }} z_{ij}$$

where $z_{ij}$ is the measurement at the $i$th location from the $j$th examination of an eye, and $N_{e}$ is the total number of examinations for the eye.

We estimated interpolation accuracy with leave-one-out cross-validation (LOOCV). In LOOCV, a set of residual errors is produced at each grid location. Traditionally, the error at the $k$th location is $e_{k} = \hat{z}_{k} - z_{k}$ where $\hat{z}_{k}$ is the result of interpolating the $k$th location from the $N - 1$ points in $\left\{ {\left( {x_{i} ,y_{i} ,z_{i} } \right)} \right\}_{i = 1,i \ne k}^{N}$, the examination data subset after removal of the $k$th point [24]. However, we are more interested in the interpolator’s ability to recover the true value from the set of measurements because this is a better indicator of accuracy between the grid locations where new values are being synthesized. Thus, in this study, the residual error was $e_{k} = \hat{z}_{k} - \tilde{z}_{k}$. For each examination, we computed the LOOCV residual error at all $N$ locations.

We summarized the LOOCV errors with three standard metrics: the mean absolute error (MAE), the root-mean-square error (RMSE), and Willmott’s modified index of agreement (d_1_) [28], which are given by

2 $${\text{MAE}} = \frac{1}{{N_{b} }}\mathop \sum \limits_{k = 0}^{{N_{b} }} \left| {e_{k} } \right|,$$

3 $${\text{RMSE}} = \sqrt {\frac{1}{{N_{b} }}\sum\nolimits_{k = 0}^{{N_{b} }} {e_{k}^{2} } } ,$$

and

4 $${\text{d}}_{1} = 1 - \frac{{\mathop \sum \nolimits_{k = 0}^{N} \left| {e_{k} } \right|}}{{\mathop \sum \nolimits_{k = 0}^{{N_{b} }} \left[ {\left| {\tilde{z}_{k} - \bar{\tilde{z}}} \right| + \left| {\hat{z}_{k} - \bar{\tilde{z}}} \right|} \right]}}.$$

Each metric was evaluated with the $N_{b}$ points, remaining after automatic detection and removal of data points in the blind spot [2, 27], which would otherwise dominate the results. Here,

5 $$\bar{\tilde{z}} = \frac{1}{{N_{b} }}\mathop \sum \limits_{k = 0}^{{N_{b} }} \tilde{z}_{k}$$

is the mean truth value for the examination. The RMSE is based on squared differences and is more sensitive to outliers than MAE or *d*_1_, which are based on the absolute values of differences. RMSE and MAE are unbounded metrics and have units matching those of the data values (dB). In comparison, d_1_ is dimensionless and takes values between 0 and 1, with 1 indicating perfect agreement between the interpolated values and the truth.

We also measured the smoothness of HOV surfaces created by interpolation of each examination onto a high-density uniform grid. These surfaces are plausible products that might be created for HOV visualization, generated as intermediates for further analysis and clinical trial end point computation, or for adaptive perimetric grid location strategies [5]. The high-density $N_{x} \times N_{y}$ grid spanned ±90° with $N_{x} = N_{y} = 501$ points. Prior to interpolation, we inserted boundary points to the examination data: 72 points with $z = 0$ around a circle with radius 120° to represent the lack of visual sensitivity beyond the peripheral field. Surface smoothness was assessed with first-order (TV_1_, introduced in [19]) and second-order isotropic total variation (TV_2_, derived from [18]). Denoting $h_{i,j}$ as the interpolated surface and $\varOmega$ as the set of points $\left( {i,j} \right)$ within the convex hull of the test grid,

6 $${\text{TV}}_{1} \left( h \right) = \sqrt {\frac{1}{\left| \varOmega \right|}\mathop \sum \limits_{i,j\in \varOmega } \left( {\left| {\nabla_{x} h_{i,j} } \right|^{2} + \left| {\nabla_{y} h_{i,j} } \right|^{2} } \right)}$$

and

7 $${\text{TV}}_{2} \left( h \right) = \sqrt {\frac{1}{\left| \varOmega \right|}\mathop \sum \limits_{i,j\in \varOmega } \left( {\left| {\nabla_{xx} h_{i,j} } \right|^{2} + \left| {\nabla_{yy} h_{i,j} } \right|^{2} } \right)} .$$

The first-order finite difference operators are given by

8 $$\nabla_{x} h_{i,j} = \left\{ {\begin{array}{*{20}l} {h_{i + 1,j} - h_{i,j} } \hfill & {1 \le i < N_{x} , 1 \le j \le N_{y} } \hfill \\ {h_{1,j} - h_{i,j} } \hfill & {i = N_{x} , 1 \le j \le N_{y} } \hfill \\ \end{array} } \right.$$

and

9 $$\nabla_{y} h_{i,j} = \left\{ {\begin{array}{*{20}l} {h_{i,j + 1} - h_{i,j} } \hfill & {1 \le i \le N_{x} , 1 \le j < N_{y} } \hfill \\ {h_{i,1} - h_{i,j} } \hfill & {1 \le i \le N_{x} ,\, j = N_{y} } \hfill \\ \end{array} .} \right.$$

The second-order operator along x is

10 $$\nabla_{xx} h_{i,j} = \left\{ { \begin{array}{*{20}l} {h_{{N_{x} ,j}} - 2h_{i,j} + h_{i + 1,j} } \hfill & {i = 1, 1 \le j \le N_{y} } \hfill \\ {h_{i - 1,j} - 2h_{i,j} + h_{i + 1,j} } \hfill & {1 < i < N_{x} , 1 \le j \le N_{y} } \hfill \\ {h_{i - 1,j} - 2h_{i,j} + h_{1,j} } \hfill & {i = N_{x} , 1 \le j \le N_{y} } \hfill \\ \end{array} } \right.$$

with a similar structure for $y$. For both TV_1_ and TV_2_, smaller values indicate a smoother surface. While both metrics are sensitive to edges, slopes, and transitions in the surface, TV_2_ is more indicative for undesired peakiness because the first derivative is zero at local apexes.

Statistical testing was performed in MATLAB. Due to the small sample size and lack of certainty about the data distributions, nonparametric hypothesis tests were used instead of standard *t* tests. First, one-tailed Wilcoxon signed-rank tests [12] determined whether the LOOCV performance for one interpolator was significantly better than the others, and whether the smoothness results were better than others. To account for the comparison among multiple interpolators, the significant level was decreased from 0.05 to 0.0055 (a factor of 9) via Bonferroni correction [6]. Second, two-tailed Wilcoxon rank-sum tests [12] determined whether the LOOCV performance for one interpolator was significantly different between the normal and patient subject groups. For these tests, a significance level of 0.05 was used.

## Results

**Fig. 2:**
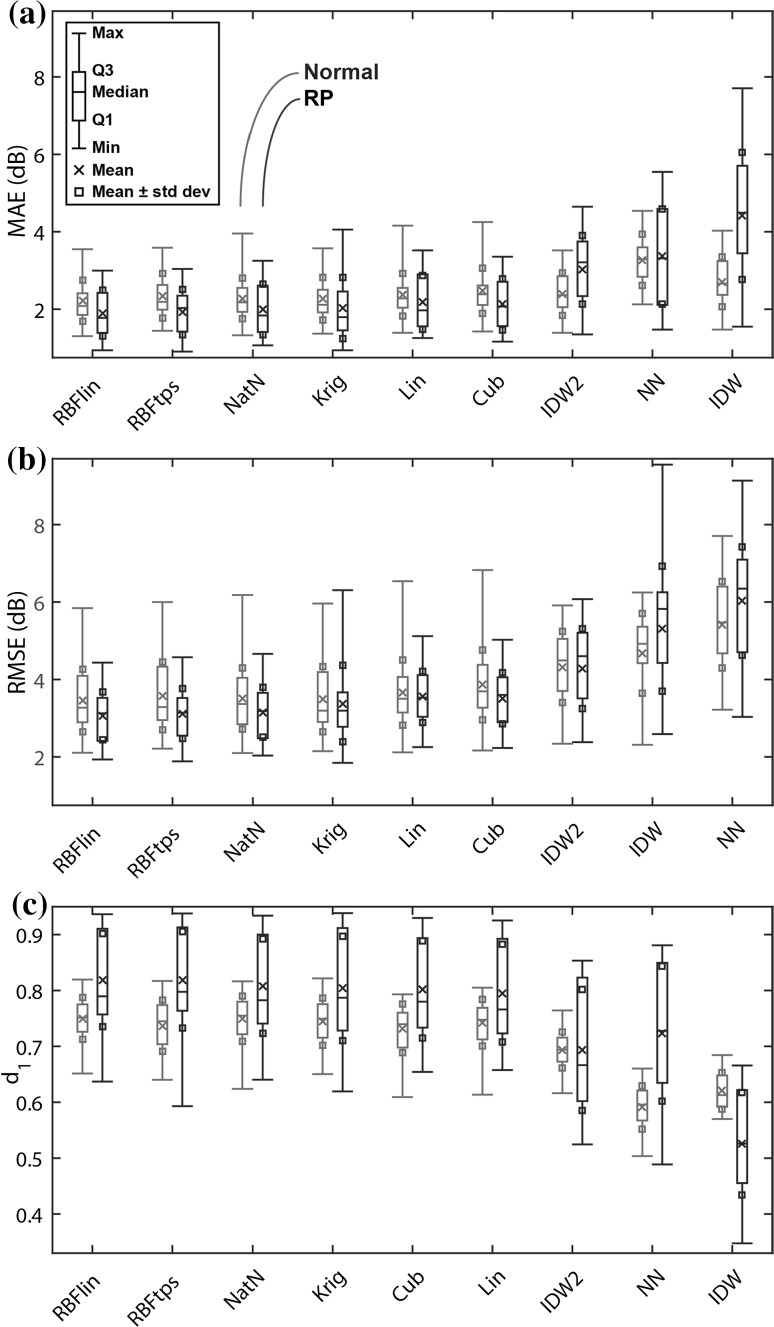
Interpolator accuracy: leave-one-out cross-validation performance as assessed by **a** mean absolute error (MAE), **b** root-mean-square error (RMSE), and **c** Willmott’s modified index of agreement (*d*_1_). In each boxplot, the interpolators are ordered left to right from best to worst performance. The linear and thin-plate spline RBF interpolators had the best performance according to all three accuracy metrics

Boxplots with basic statistical summary measures for the LOOCV-based accuracy metrics (MAE, RMSE, and *d*_1_) are presented in Fig. 2 for the normal and patient groups. For each metric, the interpolators are ordered by mean performance across all subjects. For all three accuracy metrics, the RBFlin, RBFtps, and NatN interpolators were the top three performers.

Table 3 shows the mean values of each metric for all interpolators. Values are shown for three groups: all subjects, normal only, and patients only. In terms of the three accuracy metrics, the RBFlin consistently had the best result for all groups, with the only exception being d_1_ performance in patients, where RBFlin has the second-best result. In general, the second- and third-most accurate interpolators were RBFtps and NatN respectively. Table 4 shows the results from determining whether the performance of RBFlin was significantly better than the other interpolators. It can be seen that RBFlin was significantly better in nearly all cases, with a few exceptions for comparisons with RBFtps, NatN, and Krig.

**Table 3:** Mean performance values for each interpolator

Metric	Group	RBFlin	RBFtps	NatN	Krig	Lin	Cub	IDW2	IDW	NN
MAE	Overall	2.01^a^	2.08^b^	2.10^c^	2.12	2.25	2.26	2.78	3.80	3.33
	Normal	2.22^a^	2.34	2.28^c^	2.27^b^	2.37	2.48	2.39	2.71	3.27
	Patient	1.90^a^	1.93^b^	2.00^c^	2.09	2.18	2.13	3.02	4.41	3.36
RMSE	Overall	3.20^a^	3.28^b^	3.28^c^	3.42	3.59	3.64	4.29	5.09	5.81
	Normal	3.45^a^	3.57	3.51^c^	3.49^b^	3.66	3.86	4.32	4.68	5.42
	Patient	3.06^a^	3.12^b^	3.15^c^	3.37	3.56	3.52	4.28	5.31	6.02
d_1_	Overall	0.79^a^	0.79^b^	0.79^c^	0.78	0.78	0.78	0.69	0.56	0.68
	Normal	0.75^a^	0.74^c^	0.75^b^	0.74	0.74	0.73	0.64	0.62	0.59
	Patient	0.82^b^	0.82^a^	0.81^c^	0.80	0.79	0.80	0.69	0.53	0.72
TV_1_	Overall	0.31^c^	0.33	0.34	0.41	0.41	0.38	0.30^b^	0.29^a^	1.58
	Normal	0.25^b^	0.29	0.27^c^	0.28	0.30	0.33	0.28	0.25^a^	1.22
	Patient	0.34^c^	0.36	0.38	0.48	0.47	0.41	0.31^b^	0.31^a^	1.77
TV_2_	Overall	0.036^b^	0.032^a^	0.048^c^	0.058	0.084	0.059	0.056	0.075	1.12
	Normal	0.023^c^	0.020^a^	0.029	0.021^b^	0.051	0.042	0.034	0.047	0.87
	Patient	0.044^b^	0.038^a^	0.059^c^	0.079	0.10	0.068	0.068	0.091	1.26

MAE, RMSE, and *d*_1_ are accuracy metrics. TV_1_ and TV_2_ are smoothness metrics. A lower value indicates better performance for each metric except *d*_1_, for which a larger value is better

^a^Best result

^b^Second-best result

^c^Third-best result

**Table 4:** *p* values from comparisons of RBFlin with the other interpolators

Metric	Group	RBFlin	RBFtps	NatN	Krig	Lin	Cub	IDW2	IDW	NN
MAE	Overall	–	<0.001	<0.001	0.003	<0.001	<0.001	<0.001	<0.001	<0.001
	Normal	–	<0.001	0.004	<0.001	<0.001	<0.001	<0.001	<0.001	<0.001
	Patient	–	0.04*	<0.001	0.07*	<0.001	<0.001	<0.001	<0.001	<0.001
RMSE	Overall	–	<0.001	<0.001	0.001	<0.001	<0.001	<0.001	<0.001	<0.001
	Normal	–	<0.001	0.04*	0.04*	<0.001	<0.001	<0.001	<0.001	<0.001
	Patient	–	0.02*	0.003	0.003	<0.001	<0.001	<0.001	<0.001	<0.001
d_1_	Overall	–	0.01*	<0.001	0.02*	<0.001	<0.001	<0.001	<0.001	<0.001
	Normal	–	<0.001	0.3*	<0.001	<0.001	<0.001	<0.001	<0.001	<0.001
	Patient	–	–	<0.001	0.02*	<0.001	<0.001	<0.001	<0.001	<0.001
TV_1_	Overall	–	<0.001	<0.001	<0.001	<0.001	<0.001	–	–	<0.001
	Normal	–	<0.001	<0.001	<0.001	<0.001	<0.001	<0.001	–	<0.001
	Patient	–	<0.001	<0.001	<0.001	<0.001	<0.001	–	–	<0.001
TV_2_	Overall	–	–	<0.001	0.9*	<0.001	<0.001	<0.001	<0.001	<0.001
	Normal	–	–	<0.001	–	<0.001	<0.001	<0.001	<0.001	<0.001
	Patient	–	–	<0.001	0.08*	<0.001	<0.001	<0.001	<0.001	<0.001

Because one-tailed testing was performed, p values are only shown for the pairs in which RBFlin had the better mean performance metric

* RBFlin was not significantly better at a Bonferroni-corrected significance level of 0.0055

**Fig. 3:**
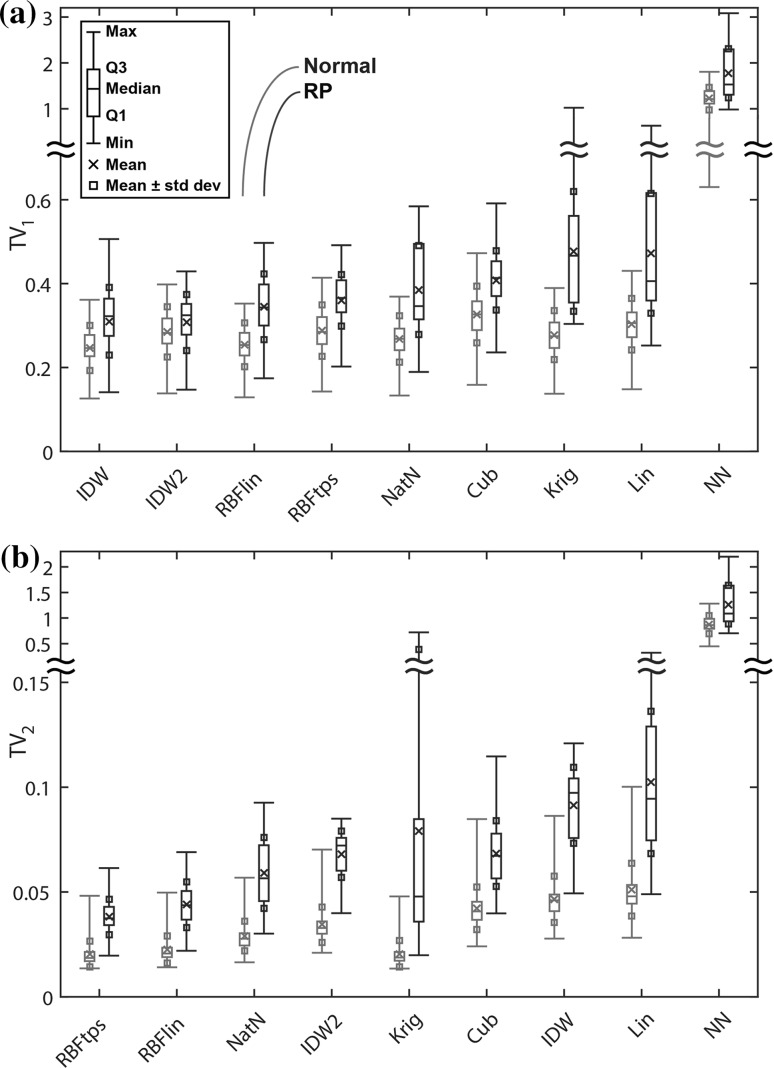
Interpolator smoothness: HOV surfaces as assessed by **a** first-order total variation (TV_1_) and **b** second-order total variation (TV_2_). Smaller values indicate more smoothness. In each boxplot, the interpolators are ordered *left to right* from most smooth to least. The thin-plate spline RBF interpolator had the best performance according to the TV_2_ metric, which is a more robust smoothness measure than TV_1_

Boxplots of the smoothness metrics are shown in Fig. 3, where again the interpolators are ordered by mean performance across all subjects. Both IDW interpolators yielded the smallest TV_1_ scores, but did not do nearly as well in terms of TV_2_. For the TV_2_ metric, RBFtps yielded the smoothest surfaces overall. Considering both TV_1_ and TV_2_, the RBFtps and RBFlin interpolators were the smoothest.

**Fig. 4:**
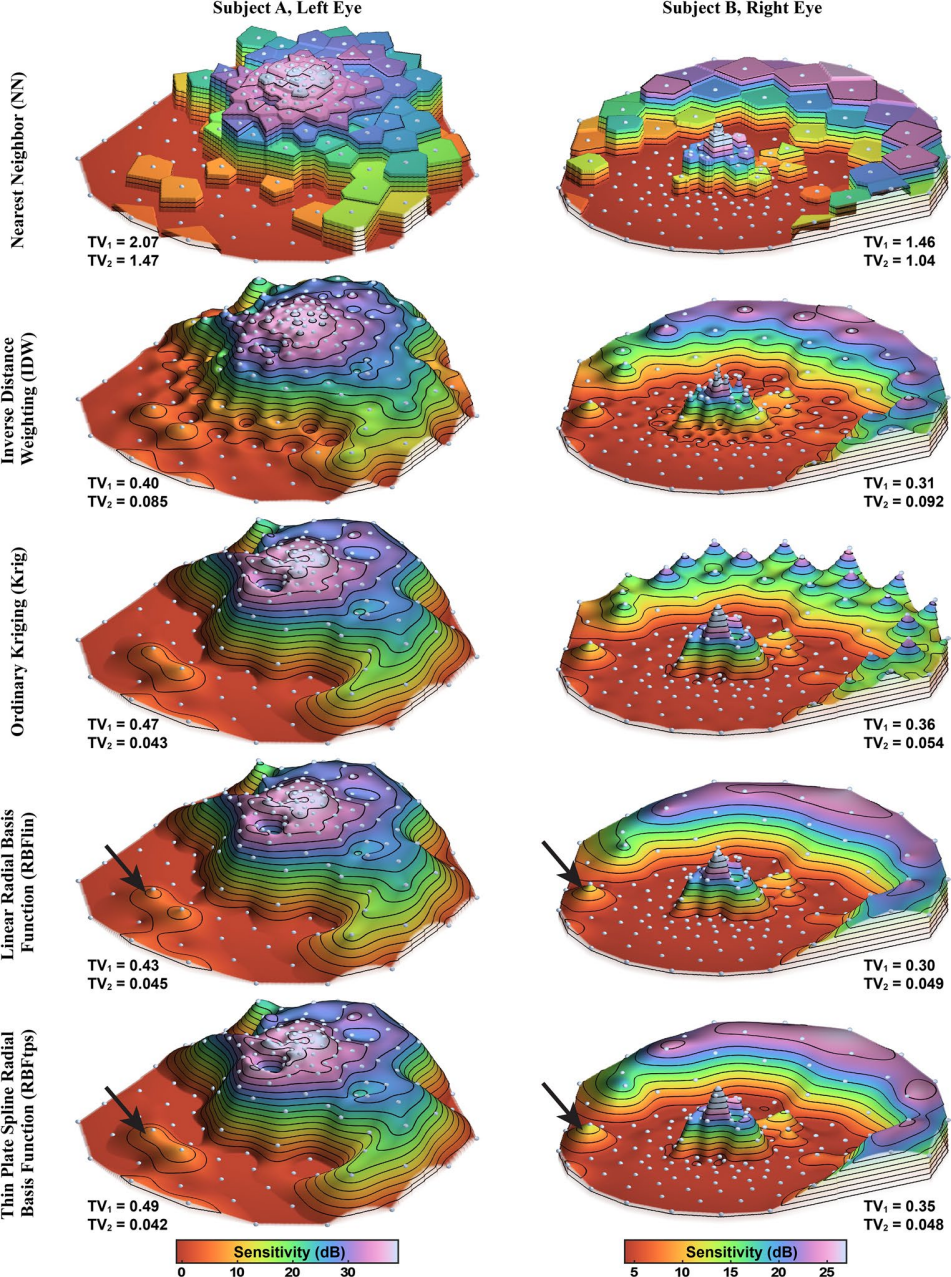
Example high-density interpolated HOV surfaces for two patients. *Higher surface height* indicates more retinal sensitivity. In each column, all surfaces share the color scale shown at the *bottom* and all iso-sensitivity *contour lines* are at the same heights. *Arrows* indicate where RBFtps is smoother than RBFlin

Visualizing the interpolated HOV surfaces helps assess the relative merit of the two smoothness metrics. Example HOV surfaces from several interpolators are shown in Fig. 4 for two RP patients. This figure illustrates the differences in smoothness characteristics that are summarized in Fig. 3. Nearest neighbor interpolation produces piecewise constant surfaces that yield large total variation scores. With IDW, significant dimpling with local peaks and valleys is apparent around the test grid locations, especially near the center where the test grid pattern is denser. The dimpling and peakiness also occurs with IDW2 (not shown). Surface TV_2_ smoothness from Kriging was highly variable among patients; the Kriging surface is smooth and almost indistinguishable from RBFtps for subject A, yet for subject B, Kriging exhibited peakiness similar to IDW2—albeit at the periphery instead of the center. The two RBF methods produced very similar surfaces, with differences only apparent around ridges and isolated rises.

Figure 4 demonstrates the importance of TV_2_ as the better metric for differentiating smoothness performance. The dimpled and peaky features of the IDW surfaces are not reflected in the TV_1_ scores. The visually smoothest surfaces are created by the RBFtps and RBFlin interpolators, and these interpolators have the best TV_2_ scores.

As seen in Table 3, RBFtps had the best TV_2_ scores for all groups. Table 5 shows the results of statistical testing of RBFtps against the other interpolators for each group. In all cases, RBFtps had significantly better TV_2_ smoothness.

**Table 5:** *p* values from comparisons of RBFtps with the other interpolators

Metric	Group	RBFlin	RBFtps	NatN	Krig	Lin	Cub	IDW2	IDW	NN
MAE	Overall	–	–	0.09*	0.9*	<0.001	<0.001	<0.001	<0.001	<0.001
	Normal	–	–	–	–	0.1*	<0.001	0.1*	<0.001	<0.001
	Patient	–	–	<0.001	0.7*	<0.001	<0.001	<0.001	<0.001	<0.001
RMSE	Overall	–	–	0.2*	0.9*	<0.001	<0.001	<0.001	<0.001	<0.001
	Normal	–	–	–	–	0.02*	<0.001	<0.001	<0.001	<0.001
	Patient	–	–	0.04*	0.06*	<0.001	<0.001	<0.001	<0.001	<0.001
d_1_	Overall	–	–	0.06*	0.9*	<0.001	<0.001	<0.001	<0.001	<0.001
	Normal	–	–	–	<0.001	0.9*	0.02*	<0.001	<0.001	<0.001
	Patient	0.06*	–	<0.001	0.09*	<0.001	<0.001	<0.001	<0.001	<0.001
TV_1_	Overall	–	–	0.9*	0.02*	<0.001	<0.001	–	–	<0.001
	Normal	–	–	–	–	<0.001	<0.001	–	–	<0.001
	Patient	–	–	0.1*	<0.001	<0.001	<0.001	–	–	<0.001
TV_2_	Overall	<0.001	–	<0.001	<0.001	<0.001	<0.001	<0.001	<0.001	<0.001
	Normal	<0.001	–	<0.001	<0.001	<0.001	<0.001	<0.001	<0.001	<0.001
	Patient	<0.001	–	<0.001	<0.001	<0.001	<0.001	<0.001	<0.001	<0.001

Because one-tailed testing was performed, *p* values are only shown for the pairs in which RBFtps had the better mean performance metric

* RBFtps was not significantly better at a Bonferroni-corrected significance level of 0.0055

Table 6 presents the significance levels from comparing the performance metrics between the normal and patient groups. For the accuracy metrics, there were significant differences between groups in most cases. For the smoothness metrics, values were significantly different between groups for every interpolator.

**Table 6:** P values from comparisons of performance metrics in normal and patient groups

Metric	RBFlin	RBFtps	NatN	Krig	Lin	Cub	IDW2	IDW	NN
MAE	0.006	0.003	0.02	0.02	0.06*	0.01	<0.001	<0.001	0.8*
RMSE	0.02	0.01	0.02	0.3*	0.7*	0.06*	0.9*	0.002	0.006
d_1_	<0.001	<0.001	0.001	0.001	0.02	<0.001	0.3*	<0.001	<0.001
TV_1_	<0.001	<0.001	<0.001	<0.001	<0.001	<0.001	0.01	<0.001	<0.001
TV_2_	<0.001	<0.001	<0.001	<0.001	<0.001	<0.001	<0.001	<0.001	<0.001

* Differences were not different at a significance level of 0.05

## Discussion

The results of this analysis are consistent with those from other reviews of interpolators for different fields of study. For example, in a study of numerous interpolators on closed-form analytic functions, Franke found that the thin-plate spline have the best scores for accuracy and visual representation [10]. Comparing RBF, IDW, and Kriging interpolators for digital terrain modeling applications, Erdogan found that the thin-plate spline RBF was the overall best performer, while IDW showed the largest errors.

Accuracy assessments with LOOCV require careful interpretation. The residual error reflects how well one data point can be recovered from its neighbors, and thus, it is proportional not only to the fidelity of the interpolator, but also to the spatial autocorrelation of the data. To isolate only the effect of the interpolator, we have focused on how the algorithms compare with one another instead of absolute error performance.

There is no practical method to obtain reliable ground truth for a visual field examination, especially in patients with progressive disease and unstable eye fixation. We constructed “truth” data by averaging data from replicate examinations under the assumption that the measurement errors are zero mean. However, this is only an estimate of the true sensitivity, which itself can change depending on patient fatigue and anxiety levels, and on the time of day [3]. True accuracy can only be gauged after the development of a realistic, full-scale visual field simulator.

In terms of accuracy and smoothness, the linear and thin-plate spline RBF interpolators yielded the best results with the latter providing slightly smoother HOV surfaces. This is reflective of the properties of the RBF kernels: the thin-plate spline kernel has a much smaller discontinuity at $r = 0$ and is infinitely differentiable everywhere else. Natural neighbor interpolation, which is available in many data analysis software packages such as MATLAB, was the next best performer. Although nominally providing a good balance between the speed of linear interpolation and the complexity of cubic interpolation, these results show natural neighbor interpolation is superior to both in terms of accuracy and smoothness.

Among the best-performing interpolators, accuracy tended to be better with RP patients than normal subjects. However, the variance was often larger in patients, meaning the accuracy performance was less consistent. This behavior is likely due to the diversity of visual field patterns in this group and the high degree of correlation present in the scotomas and low-sensitivity regions. Smoothness was significantly better in normals than in patients for every interpolator. This result is not surprising given the lack of scotomas and other visual field defects among normals that would otherwise interfere with the stability and continuity of the HOV surface.

Here, we have analyzed only a small set of interpolation techniques. We specifically chose these methods because they are relatively simple and readily available, and make good initial candidates for clinical implementation. However, we recognize that incorporating rigorous parameter estimation could improve both IDW methods and allow additional interpolators into the study including parametric RBF approaches. Also, although we did not include techniques based on approximation or regularization, we believe that developing appropriate models for these techniques will help mitigate measurement error and variability and should be the focus of future research.

There is likely no unconditionally optimal method for visual field interpolation, only a best method for a specific set of circumstances. Machine learning and algorithm portfolio optimization techniques [16] could be employed to select which interpolator or interpolator combination is best suited for a particular instance.

## Conclusion

Interpolation can be beneficial for both clinical and research analysis of static visual field data. Interpolation facilitates biomarker and clinical trial end point computation, multi-modal data alignment, and quantitative analyses that require higher sampling density. In this study of nine nonparametric methods, interpolation with linear and thin-plate spline radial basis functions yielded the best accuracy and smoothness performance. If these methods are unavailable or too difficult to implement, natural neighbor interpolation is the next best choice at a cost of reduced smoothness of interpolated surfaces.
